# Genomic Sequencing Capacity, Data Retention, and Personal Access to Raw Data in Europe

**DOI:** 10.3389/fgene.2020.00303

**Published:** 2020-05-06

**Authors:** Shaman Narayanasamy, Varvara Markina, Adrian Thorogood, Adriana Blazkova, Mahsa Shabani, Bartha M. Knoppers, Barbara Prainsack, Robert Koesters

**Affiliations:** ^1^Megeno S.A., Esch-sur-Alzette, Luxembourg; ^2^Centre of Genomics and Policy, McGill University, Montreal, QC, Canada; ^3^Faculty of Language and Literature, Humanities, Arts and Education, University of Luxembourg, Esch-sur-Alzette, Luxembourg; ^4^Metamedica, Faculty of Law and Criminology, Ghent University, Ghent, Belgium; ^5^Department of Political Science, University of Vienna, Vienna, Austria; ^6^Department of Global Health & Social Medicine, King’s College London, London, United Kingdom

**Keywords:** NGS, ELSI, policies, procedures, patient rights, research participant rights, raw, GDPR

## Abstract

Whole genome/exome sequencing (WGS/WES) has become widely adopted in research and, more recently, in clinical settings. Many hope that the information obtained from the interpretation of these data will have medical benefits for patients and—in some cases—also their biological relatives. Because of the manifold possibilities to reuse genomic data, enabling sequenced individuals to access their own raw (uninterpreted) genomic data is a highly debated issue. This paper reports some of the first empirical findings on personal genome access policies and practices. We interviewed 39 respondents, working at 33 institutions in 21 countries across Europe. These sequencing institutions generate massive amounts of WGS/WES data and represent varying organisational structures and operational models. Taken together, in total, these institutions have sequenced ∼317,259 genomes and exomes to date. Most of the sequencing institutions reported that they are able to store raw genomic data in compliance with various national regulations, although there was a lack of standardisation of storage formats. Interviewees from 12 of the 33 institutions included in our study reported that they had received requests for personal access to raw genomic data from sequenced individuals. In the absence of policies on how to process such requests, these were decided on an *ad hoc* basis; in the end, at least 28 requests were granted, while there were no reports of requests being rejected. Given the rights, interests, and liabilities at stake, it is essential that sequencing institutions adopt clear policies and processes for raw genomic data retention and personal access.

## Introduction

Whole genome sequencing (WGS) and whole exome sequencing (WES) have become widely adopted in research and, more recently, in clinical practice (Birney et al., 2017; Birney, 2019). The generated raw genomic data (i.e., WGS/WES data) include vast amounts of information of potential importance to an individual’s current and future health, with implications for family members, if analytic and interpretive hurdles can be overcome. The wide availability of genomic data also offers opportunities for reuse for additional clinical, health, research, or recreational purposes. People requesting access to their own raw data, however, raises a number of legal, ethical, and practical questions. Legally, patients in many countries have a right to access their health record (Thorogood et al., 2018). Individual access rights are also being strengthened under data privacy laws. For example, the European Union General Data Protection Regulation (GDPR; European Parliament and Council, 2016), in force since May 2018, stipulates a general right of data subjects to access their personal data. GDPR leaves it to member states to decide if and how this right applies in research contexts and to raw genomic data specifically. Given the broad translational spectrum in genomics, however, it can be difficult to clearly distinguish clinical and research contexts (Schickhardt et al., 2020). Another legal uncertainty is whether or not access rights extend to raw sequence data, though broad definitions of personal (health) data support this interpretation (Thorogood et al., 2018). There are ethical arguments for and against personal genome access.

On the one hand, some argue that research participants and patients (collectively referred to as “sequenced individuals”) have a moral right to access their own raw (uninterpreted) genomic data in both clinical and research contexts as something that fundamentally belongs to them (Nelson, 2016; Schickhardt et al., 2020). Access can also potentially empower sequenced individuals to direct the analysis and sharing of their own data, potentially improving their own knowledge and health, as well as accelerating research and innovation (Lunshof et al., 2014; den Dunnen, 2015; Middleton et al., 2015; Wright et al., 2017, 2019; Shabani et al., 2018; Thorogood et al., 2018). Providing data may also be a way of encouraging and engaging participants in research (Middleton et al., 2015). On the other hand, some express concerns that providing personal access is at best pointless and at worse harmful for individuals and burdensome for providers and health systems (Bredenoord et al., 2011). Individuals may not be able to do anything with the genomic data, or they may misinterpret the data. This is especially true if the data are of uncertain quality, as is often the case in research contexts. They may share it with unscrupulous researchers or unregulated service providers, exposing them to further misinterpretations and privacy harms (Guerrini et al., 2019). Of course, some of these risks can be mitigated through clear policies, oversight, education, and access to counseling services (Shabani et al., 2018; Schickhardt et al., 2020). However, this, in turn, raises practical resource questions, especially for research projects. Moreover, policies, processes, and infrastructure are required to sustainably manage and transfer large raw genomic data formats (Middleton et al., 2015; Wright et al., 2017).

To better understand current practices of personal access to raw genomic data by sequenced individuals, we conducted interviews with genomics professionals working in institutions within the EU/EEA that routinely perform WGS/WES of human individuals on a large scale (i.e., “sequencing institutions”). Sequencing institutions can be viewed as gatekeepers or enablers for sequenced individuals in accessing their personal raw genomic data. Furthermore, owing to their geographical location and/or the data they use, these institutions are expected to be directly impacted by the GDPR, which makes their practices particularly insightful and timely in light of the evolving regulatory landscape. For uninitiated readers, the following primer describes the impact of the GDPR on health research: Dove (2018). This study is the first to provide empirical insights into the policies, practices, and perspectives within sequencing institutions pertaining to individual access to raw genomic data. We also consider technical aspects of sequencing capacity and data retention practices, as these variables determine the overall availability of data. Our findings provide valuable empirical observations that can inform legal and ethical debates over personal genomic access, and indicate practical and technical solutions for sequencing institutions seeking to respond to such requests.

## Materials and Methods

### Interview Guide

A semi-structured interview guide was prepared consisting of questions pertaining to practices around WES/WGS, with a particular focus on genomic data retention and provision of access to sequenced individuals. The interview guide included both closed-ended questions (aimed primarily at describing the profile and practices of the sequencing centres) and open-ended questions, intended to gauge respondents’ attitudes toward a specific issue. To ensure a clear and intuitive structure of interviews, we divided the interview guide into five distinct sections (“modules”) addressing the following topics: (i) organisational structure of the institution; (ii) sequencing throughput and capacity; (iii) data management and storage capacity; (iv) data retention policies and access policies for sequenced individuals (to their own data); and (v) sequencing centres’ experiences with receiving requests from individuals to access their raw genomic data.

The draft versions of the interview guide underwent multiple rounds of internal review and refinement. The final round of refinement was carried out upon receiving feedback from the first 10 interviews within the study. The interview guide is available as Supplementary File 1.

### Identification of Sequencing Institutions

This interview study specifically targeted institutions located in the EU/EEA that were member states of the EU/EEA, and that generate, process, and/or manage human WGS/WES data for research and/or clinical purposes. As such, we refer to such institutions as “sequencing institutions.”

In order to identify sequencing institutions, we used all of the following methods: (i) web searches, (ii) prior knowledge, (iii) peer recommendations, (iv) media articles or announcements, and (v) personal relationships. Our research strategy identified 83 sequencing institutions from 23 member states across the EU/EEA region. Sequencing institutions were approached individually with the request to participate in the study.

### Participant Recruitment

Recruitment of participants started in May 2018 and continued in parallel to interviews with early respondents. A total of 64 sequencing institutions, out of a possible 83, were invited to participate in the study via email, of which 33 eventually agreed. Interviews took place between June 2018 and April 2019. Individual respondents who participated in our study were all affiliated with sequencing institutions, within which they held various positions and responsibilities. Three interviews included multiple respondents, with a maximum of five respondents from the same institution being in the same interview. This brings the total number of respondents to 39 (Supplementary File 2: Table S1). Recruitment ended when it became clear from the interviews that data saturation had been reached and no new insights were emerging from additional interviews.

### Pre-interview Communication

Respondents (representing sequencing institutions) who agreed to take part in the interview study were provided with information about the purpose of the interview, the thematic areas of focus, and the methods (Supplementary File 3). Potential respondents were also provided with a confidentiality statement explaining how the collected data would be treated (Supplementary File 4). Additional measures for data privacy, confidentiality, and security are detailed within Supplementary File 5.

### Semi-Structured Interviews and Recordings

Interviews were carried out either with Zoom video conferencing software^1^ or in person. At the beginning of the interviews, the interviewers briefly described the interview process and addressed any issues of confidentiality and privacy of the respondent (i.e., Supplementary Files 4, 5). The conversation then proceeded with personal introductions by the interviewers and respondents after which the interviews progressed according to the interview guide (Supplementary File 1). At the end of the interviews, the respondents were asked to provide concluding remarks or suggestions, if any, for further improving the interview guide.

### Transcription and Review

Interviews were transcribed using otter.ai^2^, an automated, artificial intelligence-based transcription software. The automatically generated transcripts were reviewed for accuracy and manually edited to correct any discrepancies with the corresponding audio files. Reviewing and editing of transcripts were carried out by VM/AB/SN and verified by RK for validation purposes.

The process of transcription was combined with the generation of interview summary documents called “review sheets” (Supplementary File 6). For each review sheet, a set of the most relevant quantitative and qualitative information, deemed to best reflect the respondent’s views, were selected from the corresponding interview transcript. The quotes were accompanied by a concise written summary of the interview. Subsequently, the review sheets were sent to the pertinent respondents who had an opportunity to comment on the document and suggest revisions if required.

### Collection and Analysis of Informed Consent Forms From Sequencing Institutions

In addition to the data collected for the primary research (i.e., semi-structured interviews), samples of informed consent forms that sequencing institutions use to consent sequenced individuals were requested via email (from the respondent) or accessed online, depending on their availability. We only collected informed consent forms from the sequencing institutions involved in the study.

### Data Curation and Analyses

The process of data analysis was divided into two parts, corresponding to the nature of the data being analysed (quantitative and qualitative data analyses).

#### Quantitative Data Analysis and Visualisation

Quantitative data analysis was applied to questions requiring numerical responses (e.g., no. of people sequenced), binary answers (yes/no), or categorical variables (e.g., purchase year of first Novaseq) from the interview transcripts that were tabulated (Supplementary File 2: Tables S1–S6). The conversion of the relevant information into the aforementioned format was performed by AB and VM. The process was independently repeated and refined by SN. Quantitative data analysis was carried out using Google Sheets, as part of Google Suite, and R statistical package (R Core Team, 2013). Data visualisation was performed using ggplot2 graphical package (Wickham, 2016). Diagrams, drawings, and schemes were generated using either Google Slides (as part of Google Suite) or Adobe Illustrator. All visualisations were refined using Adobe illustrator.

#### Qualitative Data Analysis

To analyse qualitative data collected through this study, we employed deductive content analysis. In this approach, themes or common content categories are pre-determined before data analysis is undertaken, as opposed to being identified in the course of data analysis (Graneheim and Lundman, 2004). Qualitative data were organised under five broad themes, which reflected the overall structure of the interview guide. The process of organising the qualitative data under these themes was undertaken by SN, AB, and VM and subsequently reviewed and validated by RK.

Interview transcripts were carefully read to identify quotes referring to one or more of the predetermined themes. The relevant quotes were subsequently placed under the most suitable theme. Quotes that bore relevance to more than one theme were divided into multiple parts and the resultant sub-quotes were placed under the suitable themes. Selection and categorisation of the relevant quotes was performed by AB and VM. The process was independently validated by SN. Discrepancies in categorising quotes were routinely discussed and resolved.

The analysis of the informed consent forms focused exclusively on individual access to genomic data and genomic data retention policies.

## Results

**FIGURE 1:**
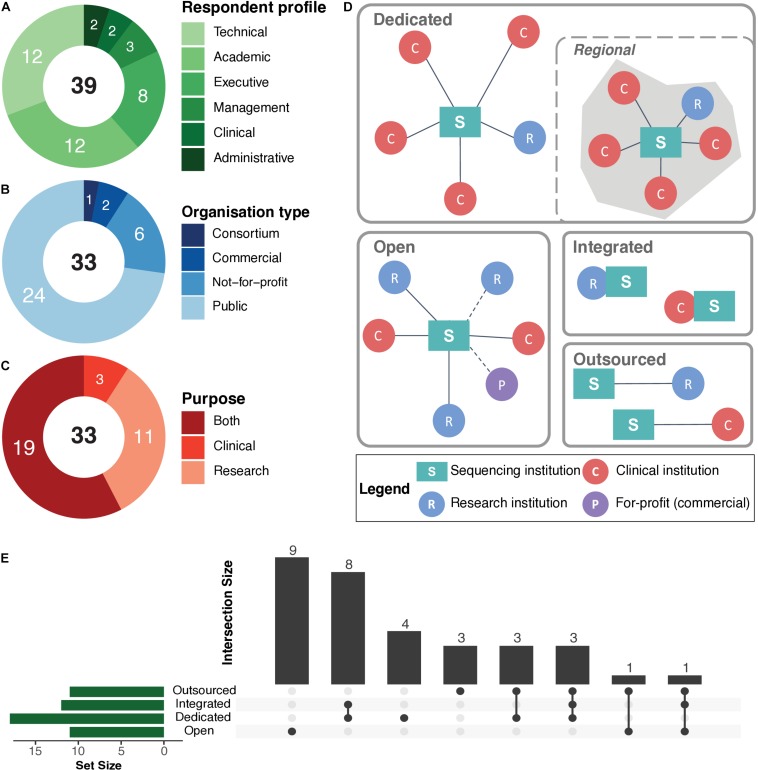
Organisation structure and operations of sequencing institutions. **(A)** Summary of respondent profiles. **(B)** Summary of organisation types. **(C)** Use cases or purpose of human whole genome (WGS) or whole exome (WES) sequencing. In **(A–C)**, numbers in black text at the centre of the rings represent the total, while the numbers in white text on the rings represent the exact numbers for a particular category. **(D)** The various operational models practiced by the institutions participating in the interview study, in terms of human whole genome and exome sequencing: “Dedicated”—operate sequencing platforms in-house to serve an exclusive set of clinical and research clients. In certain cases, such institutions serve clients within a given region/locality (i.e., “Regional”). “Open”—a standard service-oriented institution with in-house sequencing platforms. “Integrated”—institutions/departments with in-house sequencing platforms that are embedded within a research and/or clinical unit. “Outsourced”—institutions that perform sequencing with external providers. **(E)** UpSet plot (Conway et al., 2017) represents the operational models and corresponding number of sequencing institutions that utilise those models for WGS/WES. The horizontal green bars represent the total number of a given operational model. The bottom panel represents specific combinations (or intersections) of operational models. The vertical bars represent the number of sequencing institutions using those combinations of operational models. Detailed information available in Supplementary File 2: Table S2.

In total, the study included 33 interviews, conducted with 39 respondents within sequencing institutions, operating in 21 EU/EEA member states (Figure 1). They relate to more than 300,000 individuals, who underwent WGS/WES between the early 1990s to the first half of 2019. We explored current practices and policies of data management and personal raw genomic data access for sequenced individuals within these different institutions.

In line with our deductive content analysis approach, study findings were organised into the following five sections: (i) organisational structure and operational models; (ii) actual vs. potential sequencing capacity; (iii) genomic data management practices and policies; (iv) data access practices and policies; and (v) future outlook.

### Respondent Profiles, Organisational Structure, and Operational Models

The first part of the interviews served as an introduction to the respondent. Overall, the respondents held various positions and responsibilities within those sequencing institutions, including technical, academic, administrative, clinical, and management (Figure 1).

Next, we sought to obtain a better understanding of the organisational structure and the operations of sequencing institutions, as those factors may influence processes and policies for personal access to raw genomic data by sequenced individuals. In terms of organisational structure, the study included mostly (24, ∼73%) public organisations, followed by six (∼18%) not-for-profit private organisations, two (6%) commercial organisations, and one consortium. Additionally, 19 (∼58%) of the participating institutions performed sequencing for both research and clinical purposes, while 11 (∼33%) and 3 (∼9%) institutions focused exclusively on research or clinical sequencing, respectively (Figure 1). The organisations included in this study varied considerably in their size and number of personnel, with the largest and the smallest institution housing approximately 3000 and 10 staff members, respectively (average ∼450). Furthermore, a total of ∼460 personnel (average ∼17, max. ∼80, min. 3) within those organisations were dedicated towards operating sequencing platforms and data analyses and management (Supplementary File 2: Table S2).

We then asked the respondents to elaborate on various aspects of their operations, including (but not limited to) their clientele, main activities (e.g., sequencing, or data processing), their institutional arrangements (e.g., university hospital, private laboratory), and whether they were outsourcing specific tasks or processes, related to human genome sequencing.

We found that the sequencing institutions we covered typically acted as service providers to healthcare and/or research institutions, but delivered their services in different ways. In this respect, we delineated four different “operational models” to further classify the sequencing institutions as follows: (i) dedicated, (ii) open, (iii) integrated, and (iv) outsourced. Figure 1 provides an illustration and descriptions of the various operational models and their adoption among sequencing institutions included in our study. We found that most sequencing institutions provided services to an exclusive set of clients (i.e., dedicated), primarily made up of sequencing laboratories affiliated with a specific clinical or research institution, and performing sequencing services exclusively for those affiliated institutions. Affiliations are determined either through formal partnerships or based on geographical regions (i.e., regional). Other institutions performed sequencing for any internal or external clients as a standard service (i.e., open model). There were also “integrated” sequencing institutions that were physically located within the premises of a larger organisation (e.g., a university, hospital, consortia/network). Finally, there were 11 sequencing institutions that outsourced their sequencing (i.e., outsourced model) and focused entirely on data analysis and interpretation.

We found that 15 (45%) of the sequencing institutions combined at least two operational models, with the most prominent combination being the dedicated and integrated models (i.e., 8, 24%). This implies that most of the integrated sequencing institutions were dedicated to their “parent” institution (e.g., hospital, university, consortium, network). The second most frequent combination (i.e., 7, 21%) was the coupling of the outsourced model with any of the other operation models (Figure 1). Additionally, we identified two sequencing institutions operating as data hubs that did not perform any in-house sequencing, but aggregated and processed sequencing data from multiple outsourced sequencing providers.

### Actual vs. Potential Sequencing Capacity

We asked respondents about their institutions’ potential and actual (i.e., throughput) sequencing capacities. The potential sequencing capacity is defined as the theoretical maximum amount of in-house sequencing (in gigabases) a given sequencing institution can perform per annum, if they were to operate at full capacity. These numbers were calculated on the basis of publicly available information concerning the sequencing platforms used by the participating institutions (Supplementary File 2: Table S3). However, we lowered the estimates to 70% of the maximum annual capacity in order to derive more realistic assumptions, as a respondent duly noted that one should consider the capacity of a given facility, rather than the capacity of the sequencing platforms.

“[…] there are some practical problems like running [the sequencers] 24/7 and some working regulations. And that’s why we are [not] operating […]at the full capacity of the sequencers, but [rather] at full capacity of the facility […]”

Respondent 1

**FIGURE 2:**
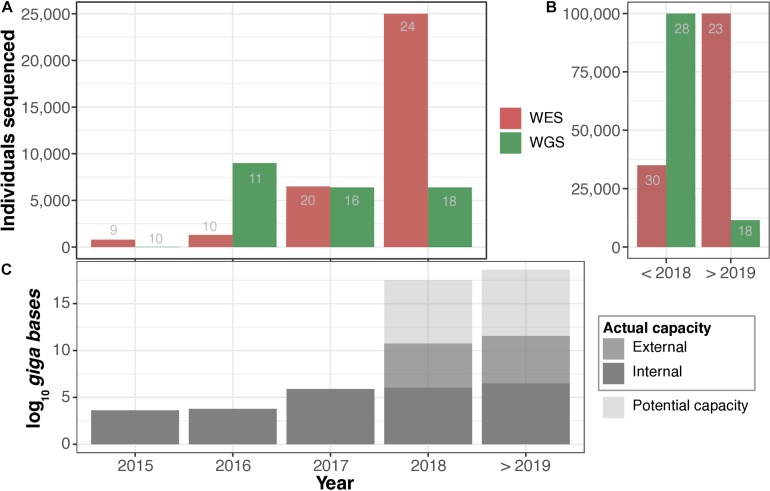
Actual vs. potential sequencing capacity. **(A)** Number of whole exomes (WES) and whole genomes (WGS) sequenced per year. **(B)** Total number of WES and WGS sequenced to date. In **(A,B)**, the number of respondents that were able to estimate the number of WES and WGS sequenced individuals are indicated in grey text within the bars. **(C)** Annual predicted (70%) potential vs. actual sequencing capacity. Detailed information available in Supplementary File 2: Tables S3, S4.

In terms of the trends and the scale of sequencing-centered activities within the participating institutions (Figure 2), collectively, the 33 institutions had sequenced ∼161,899 whole genomes and ∼155,360 whole exomes (317,259 samples in total) at the time of our interviews. The sequencing of the first WGS began in the early 1990s (by a large consortium), while the first WES was generated in 2008. The sequencing coverage (or depth) for genomes (WGS) ranged from 5× to 200× (mean = ∼51×, median = 30×) across the institutions; for exomes (WES), the sequencing coverage ranged from 10× to 300× (mean = ∼107×, median = 100×; Supplementary File 2: Table S4). The aggregated potential sequencing capacity of the institutions was approximately 1.9 × 10^7^ gigabases per year, which translates into ∼198,000 WGS per year at 30 × coverage. Based on this information, we additionally estimated the potential future scale of long-term genomic data retention.

We then asked respondents to estimate the future sequencing capacity of their institutions to help us form a clearer view on trends in WGS/WES in EU/EEA. Respondents representing 17 different institutions indicated plans for expansion, while 15 of them expressed a clear intention to purchase additional state-of-the-art sequencing platforms. Figure 2 shows the estimated combined historical and future sequencing capacity. Considering this input, we estimated that the total future potential capacity would increase to 3.0 × 10^7^ gigabases per annum, equivalent to more than 300,000 WGS at 30 × coverage. Most respondents were unable to predict actual future sequencing capacity outside of funded research projects (Supplementary File 2: Table S4). It is important to note that the reported numbers and projections are solely based on estimates provided by the respondents and are not meant to serve as accurate measures. We note, however, that business decisions to purchase sequencing machines may be an indicator of perceived future demand.

### Genomic Data Management

**FIGURE 3:**
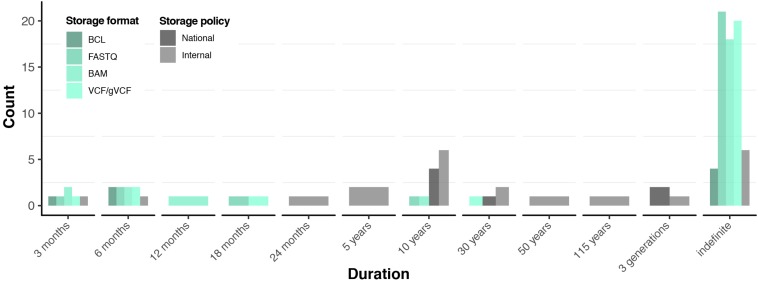
Data retention practices and policies. Green scale represents storage practices in terms of stored file formats. Grey scale represents national and internal institutional data storage policies reported by the respondents. Detailed information available in Supplementary File 2: Table S5.

The massive output from sequencing institutions generates large amounts of data and thus creates a downstream challenge in data management. When specifically addressing raw genomic data access for sequenced individuals, there were two aspects that we were interested in: (i) what is available for access in terms of raw genomic data file formats and (ii) how long will they remain accessible. Those factors, however, were constrained by (i) the capacity of the data storage infrastructure and (ii) data retention policies, which could be dictated by either internal (institutional) policies or national or EU regulations. Figure 3 shows the storage duration of raw genomic data file formats and various policies that govern retention of genomic data.

#### Practices

Discussions with respondents centred on the so-called raw file formats in the genomic data processing chain, which include (listed in order of production) the following: (i) BCL, (ii) FASTQ, (iii) BAM (including all subtypes), and (iv) VCF. It is important to note that those file formats may span up to 100 GB (for WGS data), while some of those formats may be redundant (e.g., BAM and FASTQ). As such, VCF and FASTQ formats cannot be converted back to the prior data format (BAM and BCL, respectively), potentially resulting in loss of data if those prior formats are deleted.

*“That is the benefit of storing BCLs rather than FASTQs because it’s an untouched data [*…*] when you do the demultiplexing of the BCL, and you might [*…*]. I mean, and then you [*…*] have to make a decision on how do you demultiplex? How do you get out the different reads from these BCLs, and that’s a decision that, [*…*] if you make the wrong decision, it cannot go back.”*

Respondent 2

Most respondents (26, ∼79%) were committed to at least one of the raw genomic data formats for indefinite periods (Figure 3). Particularly, the most widely retained are FASTQ (26, ∼79%), followed by both BAM and VCF (25, ∼76%), while four (12%) institutions committed to retaining BCL files indefinitely (Supplementary File 2: Table S5). In most cases, sequencing institutions maintained their own data storage (including back-ups) and computing facilities. As such, our respondents reported that a total of 284,522 (134,202 WGS and 150,320 WES) raw genomic data sets were retained by the sequencing institutions, which represented ∼90% of the total number of samples sequenced to date (see the *Actual vs. potential sequencing capacity* section and Supplementary File 2: Table S5). Respondents explained that raw data formats were stored for future re-analysis and re-interpretation. When specifically asked about the sustainability of storing those files indefinitely, respondents were generally confident in their capability to manage the data in the near future (e.g., 5–10 years). This is further supported by the fact that only five institutions utilise or plan to utilise state-of-the-art genomic data compression technologies (e.g., CRAM, Hsi-Yang Fritz et al., 2011; Bonfield, 2014) to save on data storage costs (Supplementary File 2: Table S5). However, most respondents considered that indefinite storage of genomic data sets might be unsustainable long-term (e.g., >10 years).

#### Policies

The genomic data retention policies of sequencing institutions ranged from 3 months to 115 years, to indefinite storage (Figure 3). The lower end of this spectrum is typically represented by sequencing institutions that practice the service-oriented open operational model (see the *Respondent profiles, organisational structures, and operational models* section) and therefore enforce strict internal raw genomic data retention (Supplementary File 2: Table S5). Consequently, the ∼10% of those so-called “unretained” genomic data sets (Supplementary File 2: Table S4) stem from such institutions. In contrast, seven sequencing institutions assumed the responsibility of storing all the genomic data in compliance with national laws for clinical data, under the assumption that genomic data are considered as clinical data (Supplementary File 2: Table S5). Moreover, those sequencing institutions support clients from healthcare and research in managing their genomic data for the time being.

*“[…] we never removed anything, but […] in our agreements [guarantee two years of storage]. [We] are basically waiting for healthcare to establish […] long-term data archiving solutions. And when those are in place, we will start moving the data there for long-term storage, for archiving. But [any data that we have] in our hands, we will [store] for two years. But [*…*] because our collaborative customer [is not] ready, [we] have said [it is] too much [of] value to destroy it […] now, so, we keep it and if [it is] not that expensive to store on tape [*…*], we can [absorb] the cost.”*

Respondent 2

Thirteen respondents stated that their data retention policies are stated within their informed consent forms (Supplementary File 2: Table S5). Upon comparing the responses to the informed consent forms that we collected, we found that all (nine) of the informed consent forms broadly addressed data retention, with four clearly stating the duration of data retention.

Respondents were asked about the impact of the GDPR that had recently come into force in May 2018. The majority of respondents answered that necessary measures in relation to genomic data management had been in place even before the introduction of the GDPR, mostly due to existing stringent laws when dealing with personal genetic information, which includes genomic data. Only one respondent reported a change in data management strategy because of the GDPR specifically, which involved switching from the long-term storage of BCL files to FASTQ to comply with the “right to be forgotten” outlined by GDPR. Compliance with this principle, our respondent said, was not feasible using BCL files.

“[…] one of the changes [we are] doing is; [switching] from BCL to FASTQs storage, because [it is easier] to remove the individuals, if they would request that.”

Respondent 2

In summary, sequencing institutions established various measures/strategies to manage raw genomic data, in compliance with laws and regulations. The wide variation in data retention policies and practices (Figure 3) is surprising considering that most sequencing institutions face relatively similar technical challenges, organisational priorities, and presumably also relatively similar regulatory frameworks with regard to data retention and data protection.

### Raw Genomic Data Access for Sequenced Individuals

**FIGURE 4:**
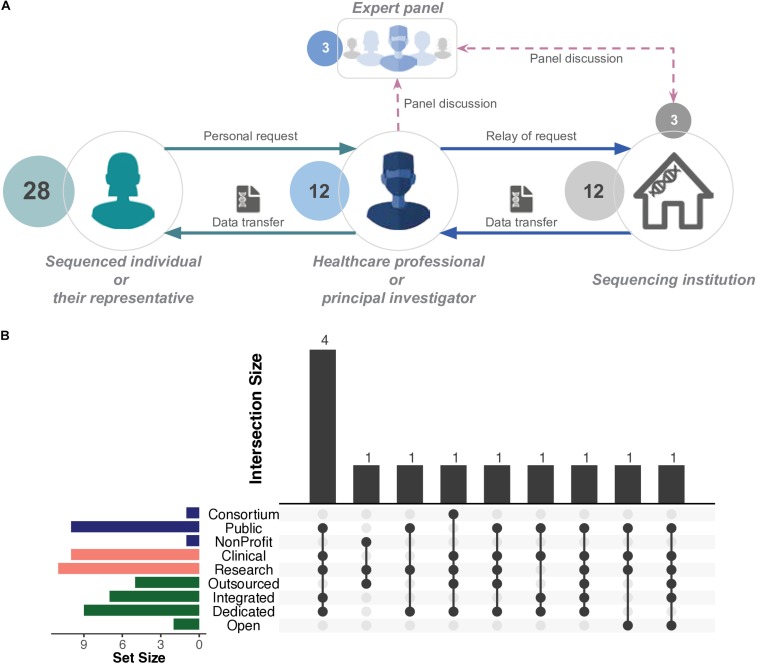
Generalised illustration of access in practice. **(A)** Typical scenario of personal whole genome (WGS) or whole exome (WES) sequencing data access. Individuals request their data through a healthcare professional or a principal investigator. Those parties authorise the access to the genomic data. In certain cases, a panel of experts, which may include personnel from a sequencing institution, jointly decide and authorise the access. The final decision is relayed to the sequencing institution, which initiates the data transfer process, mediated by a healthcare professional or principal investigator. Sequenced individuals do not directly interact with the sequencing institution. For detailed descriptions, refer to Table 1. **(B)** UpSet plot (Conway et al., 2017) represents the number and types of sequencing institutions that successfully enabled personal access to raw genomic data of patients and/or research participants. The horizontal bars represent the elements of organisational structure, purpose of sequencing, and operational model (Figure 1). The bottom panel represents the intersections between the aforementioned elements. The vertical bars represent the size of those intersections. Detailed information available in Supplementary File 2: Tables S2, S6.

In the final part of the interview, we asked the respondents if they had ever experienced cases of individuals seeking to access their raw genomic data. Accordingly, we documented at least 28 such cases (in 12 sequencing institutions within 10 countries) of sequenced individuals requesting and subsequently receiving access to their own genomic data (Figure 4). It is important to note that all sequencing institutions that received such requests ultimately granted access to the raw genomic data. We further asked those respondents who managed such cases to elaborate on how the process was carried out. We also asked all respondents about policies governing the personal raw genomic data access.

#### Practices

The most common raw file format provided to the individuals was VCF followed by BAM and FASTQ files (Supplementary File 2: Table S6). Two respondents mentioned that the sequenced individuals in question wanted their data to perform their own analyses. None of the other respondents knew the exact reasons why individuals had requested access to their own raw genomic data, but broadly speculated that those individuals were looking for second opinions.

“Well, it was actually a patient who wanted a second opinion on the data. And it was an individual, who [was] educated [in] bioinformatics, and wanted to have a look at the data [themselves] and have some second opinion about it […]”

Respondent 3

Sequenced individuals and sequencing institutions are not in direct contact; therefore, access requests are relayed through an “intermediary contact,” usually a healthcare professional, trial master, or principal investigator (Table 1). Additionally, the authorisation to grant access to a given sequenced individual appears to rest solely in the discretion of the aforementioned intermediary contact or, in some cases, is evaluated by a panel that may involve personnel from the sequencing institution, e.g., respondents themselves (Table 1). The sequencing institution (i.e., where the data resides) complies with the decision of the intermediary contact or panel and acts accordingly (Figure 4). In summary, those intermediary contacts may be viewed as gatekeepers for sequenced individuals to access their raw genomic data.

**TABLE 1:** Communication, decision, authorisation, and data transfer related to personal access to genomic data.

Communication	*“[…]. In both cases, the request came via the collaborator, [because] the individuals don’t know us. And they are not even aware that we are the ones processing their sample. […]”* *Respondent 2*
	*“[…]. Two other cases came through the actual hospital where the patients were on treatment because they came with a metastatic condition. The primary case or prior cases have been investigated with us. [*…*] the patients have been asking through the hospital, where they could get access to their data to make the most use of the old data together with the new ones out there.”* *Respondent 1*
	*“Well, […] I don’t have any direct [contact] with the individuals. It is done by the [researchers] or by healthcare.”* *Respondent 2*
	*“Yes, it’s a medical doctor, who will send us a query and he will manage all the things with this patient and [*…*], we are not allowed to send the data directly to people.”* *Respondent 7*
Decision and authorisation	*“[…] the [trial] coordinator; that’s usually the first point of contact, and then they contact for the reply of clinical heads of this program, and they decide it.”* *Respondent 1*
	*“Well, you know, since we do the service [for] other [researchers], these questions are the responsibility of the researcher and not us, except for those projects that are [for] my research groups – internal projects.”* *Respondent 4*
	*“So, it’s discussed in committees that are set up, and then it’s kind of communicated directly to the person-owner of the data.”* *Respondent 1*
	*“[…] we came to the agreement that*,[…*] this was obviously a person who was very interested and who had some prior knowledge of what [he asked for]. So, we decided on giving out both [the BAM and VCF].”* *Respondent 3*
Data transfer	*“[*…*] we just need to put them on to some external hard drive or whatever, and we hand this out.”* *Respondent 1*
	*We send the data on hard drives to the collaborator, and then they send it further to [the sequenced individuals], and we don’t know the name of individuals. We use numbers.* *Respondent 8*
	*“[…]. Right now, we had like two or three times, and we sent hard drives with the data.”* *Respondent 9*

Requests were typically handled by institutions on a case-by-case basis, using *ad hoc* procedures. Only one institution confirmed a standardised internal procedure/process to comply with such requests. Most respondents reported handing out the data to the sequenced individual on an external hard drive, while a small number were able to provide it via download. Organisations employed measures such as pseudonymisation and encryption to ensure confidentiality and security.

Four cases of personal raw genomic data access requests occurred in sequencing institutions that were public organisations with dedicated and/or integrated sequencing platforms (Figure 4 and section *Respondent profiles, organisational structures, and operational models*). The two commercial sequencing institutions (with open operational models) did not experience such requests. Furthermore, access cases within 10 institutions were linked to clinical utility (Figure 4), further highlighting the role of healthcare professionals within those cases (Table 1).

#### Policies

We compared the reported practices of the institutions by asking all respondents about their data access policies for sequenced individuals, including examples of their informed consent forms. We found that two out of nine informed consent forms provided to us included information about individual access policies. We also asked respondents if data access was granted based on a certain law or policy. In general, respondents viewed data access as a right of the individual. One respondent highlighted an organisational policy of not providing data access to minors until they are of legal age.

*“In my own projects, we have an outspoken policy that says, they are children and we are not giving up the data to them. So, when they [turn] 18, and if they ask for the data, we will ask them instead, to give a DNA sample so we will do resequencing [*…*] for them. But we are not going to give the data to them.”*

Respondent 4

Several respondents also pointed out possible contradictions between GDPR and national laws, such as the one illustrated in the next quote:

*“[*…*] the challenge that we have is that; there is actually, at some point, a contradiction between GDPR and [a national law pertaining to genetic testing]. Because, for example, we are not allowed to give genetic data [or] genetic results to the patients without [involving a] specialist, discussing the data with the patient first. So, when the patients request their data, we have to make sure that we [*… *just can’t*…*] give them the data and [say], ‘so here’s all the variants’. Even worse, if it’s a child, we cannot just give the data to the parents [and] say, okay, we’ll do whatever you want. So that is one [challenge], and we haven’t had a case yet that somebody asked for that data according to GDPR, but this is an ongoing discussion internally [on] what’s the best way [*…*].”*

Respondent 5

We also observed opposing opinions on who should bear the cost(s) associated with providing personal access to raw genomic data, e.g., infrastructure, hardware, and staff/administration. Specifically, one respondent noted that the institution will not be able to cover potential costs.

I can only say [that] the institute will not be able to pay [for the hard drives to store the raw genomic data]. [Especially], when […] people are coming [to us], requesting for the data, [but] the download speed is not fast enough, via internet. [Therefore] it is, of course, [should] not [be] the responsibility of the institution to pay [for] it.”

Respondent 6

Another respondent highlighted that it was possible for their institution to fund the associated cost(s), despite the law allowing them to reject requests if costs were too high.

*“If they want to access [to the data in] electronic format, [then] they [also have] the right to get [it] in [the] electronic format. [In a national law] there’s also a caveat that says, if the effort and the financial cost would be too high, then you can refuse the request. But we see that it’s possible [*…*].”*

Respondent 1

Finally, it is unclear if such data access is fully compliant with the right of individuals to access their health record or their personal data.

### Outlook

At the end of the interviews, we asked all the respondents to provide their future outlook on such cases of personal raw genomic data access. It must be noted that answers include both institutional policies and personal views of respondents, whereby the latter does not represent institutional policies. Twenty-one (∼64%) responses were supportive of the right and providing an option for individuals wanting to obtain access to their own sequencing data. Fourteen (∼42%) believed that the number of personal raw genomic data access requests from sequenced individuals will grow in the foreseeable future. Finally, 10 (∼30%) respondents indicated that their respective organisations were currently developing processes to manage those requests (Supplementary File 2: Table S6).

## Discussion

This study represents the first empirical study of genomic data management and personal access to raw genomic data for sequenced individuals. It demonstrates the frequency of access requests, the overwhelming tendency of sequencing institutions to grant such access, as well as technical and procedural complexities involved.

### Organisational Structure and Operational Models

This study covered a diverse set of sequencing institutions within the EU/EEA genomics ecosystem, with varying organisational structures and operational models, thus providing a good representation of the current landscape, especially in the context of evolving regulations (i.e., GDPR).

#### Accountability for Data Retention and Personal Access

The institution or department responsible for genomic data management are typically physically, if not legally, separate from the institution or department responsible for interaction with sequenced individuals. However, certain organisations had units performing both data management and communicating with sequenced individuals within the same physical location, but were in fact independent, yet highly collaborative entities (e.g., departments, units, or groups). On the other end of the spectrum were institutions within which these two functions were located in clearly distinct institutions, with a clear service-based relationship and minimal collaboration. Particularly for institutions where several functions are fulfilled by the same units, it is necessary to clarify how these functions correspond with roles and responsibilities that are of legal relevance. The GDPR, for example, distinguishes between “data controllers,” who determine the purpose of processing and who are primarily responsible for respecting individual rights, and “data processors,” who carry out data processing services on behalf of controllers. The GDPR also recognises the possibility of joint controllership, where more than one party is responsible for protecting data and meeting demands from the individual right of access.

In light of these complex organisational structures, we recommend that there should be clear instructions for individuals regarding how to request access to their raw genomic data, including a clear point of contact, when they consent to having their genome sequenced. If requests for an individual’s raw genomic data access are made directly to the sequencing institution/unit, they may need to be directed back to the appropriate access point. It should also be clear which organisation is responsible for determining if access should be provided, and according to what criteria. A failure to respond to access requests under the GDPR could lead to legal liability for both the requesting party and the sequencing institution.

#### Clinical Versus Research Data

One important consideration in discussion about both data retention and right to access to raw genomic data is the distinction between research and clinical data. Most notably, research data may not be considered of sufficiently high quality to enable meaningful consumer reuse (Shevchenko and Bale, 2016). Consumers may insist, however, that it is them, and not the sequencing institution, who should be able to make this determination, if necessary under the guidance of relevant experts (e.g., genetic counselors). In Europe, however, the right of data subjects to access their own raw genomic data in the research context may be restricted by member states, under Article 89 of the GDPR. While access rights are typical in the clinical setting, it remains unclear whether or not raw genomic data are considered part of patients’ medical records (Thorogood et al., 2018). Even if there is no legal requirement, research projects may still opt for ethical or engagement reasons to provide access. Complicating things further is that a significant number of sequencing institutions provide both types of sequencing (research and clinical). The emergence of numerous national clinical genomics projects designed as learning health systems that routinely collect clinical data for the purposes of both care and research is also eroding this distinction (Stark et al., 2018; Price and Cohen, 2019).

Sequencing institutions may need processes to distinguish between research and clinical data for the purposes of retention or personal access. Alternatively, they may decide to adopt a single policy on personal access for all data in favor of the strictest requirements (i.e., to provide access).

### Actual and Potential Sequencing Capacity

Our respondents reported rapid increases in WES/WGS potential and actual sequencing capacity from previous years. Moreover, growing competition between manufacturers in producing the most cost-efficient sequencing technology platforms was seen by our respondents as providing sequencing institutions with ample choices for further expansion of their sequencing capacity, and thus leading them to “stock up” on sequencing capacity. It must be noted that the unused sequencing capacity is typically due to various limiting factors such as (i) research funding, (ii) capacity of facility/institution, (iii) consortia activity, and (iv) clinical demand. The latter is especially true as respondents could not predict future actual capacity related to clinical genomic data, likely because of (i) unclear public healthcare allocation (budget) for WES/WES, (ii) emerging state-of-the-art sequencing platforms, which may result in (iii) falling costs of WES/WGS. Yet, sequencing institutions foresee performing more WES in the near future as it currently is and it will be considerably cheaper compared to WGS, indicating that the lowering costs of WGS is still insufficient to justify its cost for all use cases, though it will be important in certain niches (e.g., rare diseases).

The increasing amounts of genomic data produced in the clinical and in the research domain will have important ramifications for both data retention and the provision of raw genomic data access to sequenced individuals. WES/WGS is a platform technology, which generates rich and stable information that can be used for multiple clinical, research, and recreational purposes over time. Reuse of sequences has potential value not only for sequenced individuals, but also for healthcare systems, science, and commerce. Of course, reuse of data depends on deployment of standard sequencing platforms, analysis pipelines (where applicable), and file formats to ensure both interoperability and quality. Our results suggest significant variation in sequencing practices and pipelines. High interoperability and quality standards are needed to ensure that sequenced individuals can access raw genomic data for consumer use or for redistribution to other service providers and researchers (patient-centric data sharing; Kish and Topol, 2015). This is to ensure that data are meaningful and trustworthy for a number of downstream, distributed users. The right of consumer portability (closely related to the “right to access”) has been recognised by GDPR by stating that data controllers should provide personal data “in a structured, commonly used, machine-readable and interoperable format” (Recital 68). While there does already appear to be a relatively high level of standardisation and reproducibility for human WGS/WES data generation and processing to enable medical and research reuse (DePristo et al., 2011; Auwera et al., 2013), most sequencing institutions do not currently provide levels of standardisation aiming to enable meaningful consumer reuse. This may change with the growing frequency and awareness of personal access to raw genomic data.

### Genomic Data Management

With the rapid increase in sequencing capacity, questions arise as to who will store raw genomic data, in what form, and for how long. Our study, the first of its kind to review data retention, reveals uncertainty over who was responsible for storing data. In some cases, sequencing institutions were storing data as a stop-gap measure until requesting organisations developed sufficient capacity to do so. There was also a general lack of clear institutional policies about the duration of data retention, and significant variation between the policies that do exist (from a couple of months to indefinite). Unclear and varied retention policies are surprising considering legal requirements of data retention that may apply, particularly in clinical contexts. Retention policies were also not consistently described in the consent forms we reviewed. This is in line with the findings of previous reviews (Shabani et al., 2018). On the basis of these findings, we recommend that sequenced individuals should be provided transparent information about the length and location of storage at the time of consenting to their DNA being sequenced. A further area for exploration would be to determine if these requirements apply, or should apply, to raw genomic data.

Data retention practices present important challenges. On the one hand, longer-term storage of data can provide practical opportunities for quality control, re-interpretation, and reuse for secondary research purposes, and also allows individuals a greater span of time to request personal genome access. On the other hand, given the potential increase in sequencing capacity, storage may soon start to pose a bottleneck and sustainability challenge (especially as we move to WGS), though respondents did not suggest this was an immediate problem. In the view of advancements in sequencing technologies and the decreasing costs of sequencing, long-term storage of data may not seem cost-efficient. Moreover, data privacy principles such as data minimisation, which dictates that personal data should only be kept as long as necessary to carry out a specific purpose, could pose challenges to long-term storage of genomic data. The implementation of such principles into practice is still an ongoing process among many sequencing institutions, as also highlighted in our study. Finding ways to ensure compliance with emerging regulatory requirements without giving up the benefits of long-term genomic data retention is one of the key challenges currently facing sequencing institutions in the EU/EEA (Wagner et al., 2014). Sequencing institutions could be supported in balancing these interests through the development of standard storage technologies (e.g., compressed file formats, electronic health records) and practices. This could be pursued initially through voluntary standards organisations (e.g., Global Alliance for Genomics and Health, GA4GH; Health Level Seven, HL7) and through professional guidelines and best practices (e.g., American College of Medical Genetics and Genomics, ACMG; Advancing Human Genetics & Genomics, ASHG; The European Society of Human Genetics, ESHG), and could eventually be incorporated into laboratory regulations (Botkin et al., 2015; Deignan et al., 2019).

That said, in determining the period for data retention in the context of raw genomic data, other existing relevant regulations, such as those concerning minimum/maximum length for storage of medical information in the healthcare setting may apply. Consequently, it would be important to clarify the status of raw genomic data, namely, whether they would be considered as part of patient medical records or not.

### Requests to Access Personal Genomic Data

Previous work has found that individuals are typically interested in obtaining access to their own genomic/genetic data (Lunshof et al., 2014; Middleton et al., 2015). It may help that various national regulations and the GDPR require data controllers to inform data subjects of their right to access data [Article 13(2)(b)]. Moreover, as more third-party service providers emerge, rising consumer awareness may lead to more individuals requesting access.

At present, however, the overall number of requests for genomic data is very modest in comparison to the number of sequenced individuals. Possible reasons for this are the relatively recent adoption of WES/WGS, as well as low interest, awareness, and consumer utility. Moreover, many genomic data sets in the EU/EEA are currently generated in research contexts that anonymise data, thus precluding return. The plausibility of this explanation is supported by our finding that only two access requests were related to research genomic data. Moreover, the complex structures and operations of sequencing institutions may lead to a lack of clear coordination of responsibility within and between organisations. For instance, service-oriented commercial sequencing institutions did not see any access cases possibly due to the lack or limited communication between such sequencing institutions and the intermediary contact (i.e., clients), beyond data generation and processing. Finally, most of the informed consent forms we analysed do not consistently mention access rights, as addressed by previous work on this topic (Shabani et al., 2018).

Our study found that in all cases where access was requested, the sequencing institution gave them access, despite a lack of formal internal policies and procedures. This is a general indication that sequencing institutions recognise their ethical responsibility and the rights (legal) of sequenced individuals to access their raw genomic data. It is therefore important for sequencing institutions to establish clear policies and procedures for personal raw genomic data access. As such, one noteworthy finding of this study is the observation that most sequencing institutions make decisions to grant access on an *ad hoc*, case-by-case basis. It is unclear who within the institution authorises the access and according to what criteria. Similarly, there are no standards or best-practice guidelines for protecting the privacy, security, and well-being of sequenced individuals during the access process. Provision of access through an intermediary with appropriate genetics expertise can help sequenced individuals better understand the meaning and limits of genetic data. It appears that the interaction between research and healthcare personnel is quite common in sequencing units integrated within healthcare institutions. Staff of dedicated and/or integrated sequencing institutions (e.g., respondents) are able to communicate directly with respective intermediary contacts, which are, in turn, able to communicate with the patient, thus creating a conducive environment for providing raw genomic data access to sequenced individuals. However, present *ad hoc* and case-by-case-based practices may not be scalable.

Once institutional policy about when to provide personal genome access has been formulated, there are additional technical and practical questions about how data will be accessed. Technically, should data be provided on a hard disk, through a web portal, or through the cloud? Who must bear the cost for such access, the individual, the requesting institution, the sequencing institution, or the healthcare system? Security and privacy measures are also important elements that need to be adequately protected when retaining, sharing, and accessing sensitive data.

It should be noted that, as of yet, there is no evidence available on what individuals do, or intend to do, with their raw genomic data after access, and it is a matter of an ongoing debate how much support they should receive in understanding/interpretation of such data, and from whom. Seeking a second opinion might be one reason for patients to request access to their raw genomic data. Currently, there are some third-party online services that also offer interpretation services to the individuals (Guerrini et al., 2019). They may also opt to share their data with interested third parties such as biotech or pharma companies in exchange for monetary or non-financial incentives (Ahmed and Shabani, 2019). However, it is not clear if patients should receive professional support when using such online services. This will, of course, depend on the context—is the interpretation for healthcare purposes (e.g., serious disease predispositions), or for more general preventative, well-being or recreational purposes? If individuals are seeking medical interpretation, the best option for individuals would be to reuse their raw genomic data within third-party healthcare institutions, which includes the guidance of qualified professionals by design (Wright et al., 2017; Middleton, 2018). However, to the best of our knowledge, this particular use case of interoperability between EU/EEA healthcare institutions is yet to be explored and may be highly complex given varying infrastructure, resources, and capabilities of different healthcare institutions. Most importantly, the aforementioned uncertainties of third-party reuse of raw genomic data should first be explored through empirical research to distinguish the concrete needs and risks from hypothetical ones (Middleton, 2018). This should further guide the development of interoperability channels specific to the reuse of genomic data.

### Limitations

A general limitation to this study is that we may not have covered all possible types of sequencing institutions (e.g., commercial institutions and consortia) and personal access requests, including those that were possibly overlooked, did not respond, or declined to participate. Furthermore, we did not cover ordering institutions (clients) that may have received access requests that were not passed on to the sequencing institutions in the study, but rather handled by the clients themselves. Most importantly, given the complexity of organisational relationships and structures, we were unable to directly interview the organisation or the gatekeepers of access requests, including healthcare professionals and expert panels. Neither did we interview those persons within an organisation most knowledgeable about its infrastructure (e.g., IT specialist) and policies (e.g., lawyer or data steward) as this was not a criterion for the selection of interviewees (we spoke to whoever from the organisation that agreed to speak with us).

We would also like to highlight potential bias between respondent sequencing institutions and those declining to be interviewed. It may be that institutions within our professional networks were more likely to agree to interview. Decliners can be characterised as follows: most of the negative responses stemmed from people who did not respond to our communication at all or failed to schedule an interview, while five outright declined to interview, with four of them providing reasons for rejecting (see Supplementary File 5). If given, the most common reason for rejection was due to the preference in answering the questions in a written format. However, we decided against it (i.e., written questionnaires) to maintain consistency of our data collection methodology and also due to the nature of the open-ended questions, which are more suited within an interview setting. However, given that we successfully surveyed a large proportion of identified institutions (63 of 83), it is likely that we achieved saturation and so this bias is expected to be limited.

We also did not systematically analyse differences in national regulatory frameworks, written institutional policies, and governance documents (if they exist), outside the limited number of informed consent forms. In that regard, we were unable to compare information from statements within the interviews with a complete set of informed consent forms from the organisations, outside the limited number of informed consent forms obtained from those organisations, used for validation. It is also crucial to investigate other potential professional concerns from the perspectives of the healthcare professionals that may disfavor personal access to raw genomic data. Future research may also want to consider cross-country comparisons of sequencing institution structure, data retention, and personal access. Our exploratory study aimed at identifying general trends rather than making these granular comparisons. Moreover, our central finding, that few institutions have formally addressed retention or personal access, seems to have general implications across Europe. Our study remains the first exploratory study providing empirical evidence on the organisational structures, current and future sequencing capacity, and approaches to genomic data retention and personal genome access of sequencing institutions located in the EU/EEA.

### Outlook

We find that sequencing capacity in Europe is growing and that some sequenced individuals are requesting access to their raw genomic data. Despite these trends, we also find that sequencing institutions are largely unprepared to handle questions of retention and personal access, and have yet to develop clear policies and practices. In a broader context, this study gives insight into the complexity and the general direction of the emerging genomics ecosystem. In that regard, we hope that this study becomes a catalyst for future explorations of similar nature with other stakeholders of the genomics ecosystem, and enhances the development policies and best practices in the context of personal access to raw genomic data.

## Data Availability Statement

The data sets generated and analysed for this study are included as part of the manuscript as Supplementary Files 1–6. Code for analysis and visualisations are available in GitHub repository (https://github.com/shaman-narayanasamy/sequencing_institution_analysis). Please contact authors for in case of any inquiries about the raw data.

## Ethics Statement

The studies involving human participants were reviewed and approved by National Data Protection Commission of Luxembourg. Written informed consent for participation was not required for this study in accordance with the national legislation and the institutional requirements.

## Author Contributions

SN and RK conceptualised and designed the study. AT, BP, BK, and MS provided critical feedback on the study design. SN and VM coordinated the study. VM, SN, and RK prepared the confidentiality statement, pre- and post-interview material. SN, VM, and RK performed the interviews (i.e., data collection). RK and VM managed communication with respondents before and after the interviews. VM, AB, and SN performed the transcriptions. SN, VM, AB, and RK carried out the data curation, analysis, and visualisation. AT, MS, BK, and BP interpreted the data. SN, AT, MS, VM, and AB drafted the manuscript while critical revision was provided by BK, BP, and RK. All the authors approved the manuscript for publication.

## Conflict of Interest

This study may guide the development of future products/services of Megeno S.A. SN, RK, VM, and AB are employees of Megeno S.A. AT does paid consulting work for Megeno. The remaining authors declare that the research was conducted in the absence of any commercial or financial relationships that could be construed as a potential conflict of interest. The reviewer SN declared a past co-authorship with two of the authors AT and BP to the handling Editor.

## Footnotes

1 https://zoom.us/

2 https://otter.ai/
