# Supplementary material for: Genomic Sequencing Capacity, Data Retention, and Personal Access to Raw Data in Europe
Source: Front Genet. 2020 May 6;11:303. doi: 10.3389/fgene.2020.00303 (PMC7218066; doi:10.3389/fgene.2020.00303)
Supplement: Supplementary file 1 [file Data_Sheet_1.PDF]

Module 1: Personal Profile

1. Name
2. Position
3. Institution
4. Location
5. Background/expertise

Module 2: Organisational Structure

1. Type of institution:
    a. What is the function of your institution (hospital, healthcare, research, consortium)?
    b. Are you a sequencing facility?
    c. Do you operate in clinical diagnostics or research?

2. Are you part of larger consortiums as a member or a leader?
    a. **If yes**, are you a consortium leader or member?

3. Governance (public, private, other)

4. Funding type (public, private, other)

5. Are you a for profit or non-profit organization?

6. Number of employees

Module 3: Sequencing Capacity

1. When did you **start your human whole genome/exome sequencing**?

2. What type of **sequencing platform(s)** do you currently operate for whole human genome/exome sequencing?
    a. How many of units of each platform?
    b. If possible, please specify the year of purchase:

3. Are you planning to expand your human whole genome/exome sequencing capacity?
    a. How many of units of each platform?

4. What types of human sequencing do you perform in your institute?
    a. Do you perform WGS or WES?
    b. Do you perform cancer sequencing?
    c. Do you perform rare disease sequencing?
    d. Do you sequence healthy controls?
    e. Other(s), indicate:

5. Please, describe your whole genome/exome **sequencing throughput**?
    a. Namely, the actual number of people sequenced?
    b. If possible, estimate the numbers for 2017, 2018 and 2019.
    c. Indicate the coverage you are sequencing the individuals at? For germline/tumor samples in cancer; for rare diseases, etc.

Module 4: Data Storage

1. In the whole genome/exome data processing chain (from BCL to VCF/gVCF), **which of the files formats do you store**?

2. **Where and for how long** are the different file types **stored**?

3. What are **the practical reasons for archiving** these files?

4. **Do you see storage becoming an issue in the near future?**

5. **Compression applied** to the files stored.

6. Please, describe your **storage capacity** in terms of:
    a. Number of individuals' data stored
    b. A stored unit size (in GB)

Module 5: Data Policies (Retention and access)

1. Is your **informed consent form available online**?

2. Does your institution have a data retention policy for whole genome/exome sequencing data?
    a. **if yes**
        i. Is this approach based on a specific law/policy?
            - If yes – if possible please name the law or policy
        ii. If possible, what is the reason?
        iii. Is it included in your consent?

    b. **if no,** why is there no data retention policy?

3. Does your institution have data access policy for whole genome/exome sequencing data specifically geared towards sequenced individuals and/or their physicians?
    a. **if yes**
        i. Is this approach based on a specific law/policy?
            - If yes – if possible, please name the law or policy
        ii. If possible, what is the reason?
        iii. Is it included in your consent?

   b. **if no,** why is there no data retention policy?

4. What **changes were implemented by your institution based on GDPR**, with regards to whole genome/exome sequencing?
   a. Do you have a specific erasure policy in the institution?

Module 6: Data Access Requests

1. To date, have you/your institution/your collaborators encountered cases of sequenced individuals **requesting for WGS/WES data access**?

2. **if yes**, was it successfully carried out?
   a. **if yes**,
      i. Who requested for the data?
      ii. Who authorised the access?
      iii. How was it communicated? (point of contact, channel)
      iv. Was it successfully carried out?
      v. How was the authorisation processed?
      vi. How was the process carried out?
      vii. What privacy and/or security measures do you employ when providing this access?
      viii. What exactly was asked to be accessed? (Raw data, processed data, interpreted findings)
   b. **if no**
      i. Why were you unable to provide access?
      ii. How would you respond to such a request?
      iii. What privacy and/or security measures would you employ when providing this access?

3. **if no,** is your organisation prepared/preparing for such potential requests **in the future**?
   a. **if yes:**
      i. How are they prepared/preparing for it
      ii. What privacy and/or security measures do you employ when providing this access?
   b. **if no:**
      i. How would you or your institution respond to such a request?
      ii. What privacy and/or security measures would you employ when providing this access?
