# Supplementary material for: Genomic Sequencing Capacity, Data Retention, and Personal Access to Raw Data in Europe
Source: Front Genet. 2020 May 6;11:303. doi: 10.3389/fgene.2020.00303 (PMC7218066; doi:10.3389/fgene.2020.00303)
Supplement: Supplementary file 2 [file Data_Sheet_2.PDF]

2019

SURVEY ON GENOMIC SEQUENCING IN EUROPEAN HEALTHCARE INSTITUTIONS

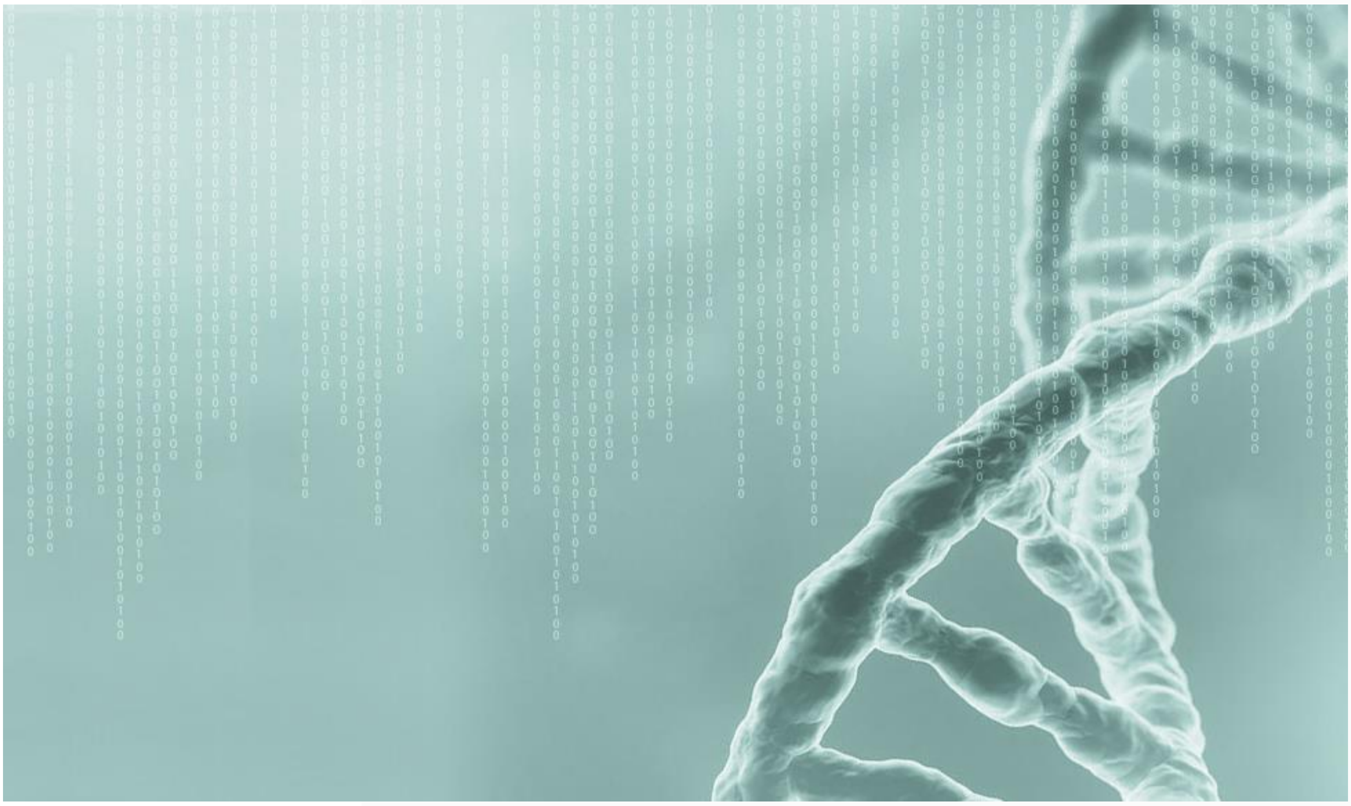

PREPARED BY MEGENO S.A.

www.megeno.com
+352 20 60 63 101
info@megeno.com

6A, AVENUE DES HAUTS-FOURNEAUX
L-4362 ESCH-SUR-ALZETTE
LUXEMBOURG

The Survey

MeGeno is conducting a Europe-wide survey specifically aimed at healthcare and research institutions that perform and interpret human whole genome/exome sequencing.

The Goal

The survey will collect information to assess current policies for whole genome/exome sequence data retention and access. We also cover aspects of organisational structure and technical capabilities. We aim to understand how emerging ethical implications and legal requirements are being implemented by European sequencing and healthcare institutions.

Your Contribution

Your participation and input is very important to us due to the currently limited number of European institutions generating whole genome/exome data. Furthermore, it helps us to understand current practices and unmet needs related to data retention and access policies. You and your institution will remain anonymous in any reporting.

Your Benefits

Your responses will be returned to you for review prior to the analysis and will remain confidential. Subsequently, you will receive summary results of the survey which will provide you with a deep insight into data retention and access policies implemented across Europe.

MEGENO

Module Overview

1. Personal Profile
2. Organisational Structure
3. Sequencing Capacity
4. Data Storage
5. Data Access Policy
6. Data Access Requests

You may skip questions/modules outside the scope of your activities.

Module 1: Personal Profile

1. Name
2. Position
3. Institution
4. Location
5. Background/expertise

NOTE: This information will remain confidential.

Module 2: Organisational Structure

1. Type of institution:
    - Sequencing facility
    - Clinical
    - Research
    - Consortium
    - Other
2. Governance (can be a combination):
    - Public/Private/Other
    - Consortium leader/Consortium member
3. Funding:
    - Public/Private/Other
4. Number of employees

Module 3: Sequencing Capacity

1. When did you start your human whole genome/exome sequencing?
2. What type of sequencing platform(s) do you operate for whole human genome/exome sequencing?
3. Are you planning to expand your human whole genome/exome sequencing capacity?
4. Which disease areas do you cover in the context of whole genome/exome sequencing, in 2017?
5. Please, describe your whole genome/exome sequencing throughput (number of individuals and coverage) for the following years: 2017, 2018, 2019.

Module 4: Data Storage

1. In the whole genome/exome data processing chain (from BCL to VCF/gVCF), which of the files do you store?
2. Where and for how long are the different file types stored?
3. What are the reasons for archiving these files?
4. Compression applied to the files stored.
5. Please, describe your storage capacity in terms of number of individuals' data stored and a stored unit size.

Module 5: Data Access Policy

1. Does your institution have a data retention policy for whole genome/exome sequencing data?
2. Does your institution have a data access policy for whole genome/exome sequencing data specifically geared towards sequenced individuals and/or their physicians?
3. Do you base the genomic data retention and access on specific laws/guidance/policies? Please, indicate the name of the law/guidance/policy, if possible.
4. What changes were implemented by your institution based on the GDPR, with regards to whole genome/exome sequencing?

Module 6: Data Access Requests

1. To date, have you/your institution/your collaborators ever been requested access to whole genome/exome data by the sequenced individuals and/or their physicians?
2. How is your organisation prepared/preparing for such potential requests in the future?
