# Supplementary material for: Genomic Sequencing Capacity, Data Retention, and Personal Access to Raw Data in Europe
Source: Front Genet. 2020 May 6;11:303. doi: 10.3389/fgene.2020.00303 (PMC7218066; doi:10.3389/fgene.2020.00303)
Supplement: Supplementary file 3 [file Data_Sheet_3.PDF]

Statement of Confidentiality

Issued by:
MEGENO S.A., a company registered in Luxembourg under company number R.C.S. Luxembourg B 197672 whose registered office is at 6A, avenue des Hauts-Fourneaux, L-4362 Esch-sur-Alzette, Luxembourg **(Megeno)**
(the Recipient)

Issued to:
Institutions and Individuals participating in a "SURVEY ON GENOMIC SEQUENCING IN EUROPEAN HEALTHCARE INSTITUTIONS" **(the Survey)**
(the Discloser)

This Statement of Confidentiality relates to information provided by the Discloser to the Recipient during one or more interviews surrounding the topic of whole human genome/exome sequencing in European healthcare and sequencing institutions. Megeno will collect, analyse and evaluate the answers provided by the Discloser in collaboration with external, independent scientists in the fields of bioethics and regulatory policies in genome medicine **(the Collaborators)**. Targeted outcomes of such collaboration include scientific publications on the basis of the quantitative and qualitative findings of the survey **(the Purpose)**.

Megeno and its collaborators respect the privacy of interview partners and their institutions and actively protect the confidentiality of the data collected at both the institutional and individual level.

1. This statement of confidentiality covers:

   a. **the Personal Information** (including name, title and background expertise of the Discloser)
   b. **the Institutional Information** (including name of the Institution and its location)
   c. **the Research Information** (any other data collected in the Survey)

   provided by the Discloser.
   Personal, Institutional and Research Information together hereinafter shall be considered **the Information**.

2. The Recipient undertakes to keep the Personal Information of the Discloser secure and only to disclose it to the Collaborators for the Purpose. The Personal Information shall not be disclosed to any other third party, as defined in the General Data Protection Regulation (GDPR). The Personal Information will be held in cloud-based storages or physical devices with restrictive access during the processing of the data until the Purpose is achieved. Once the Purpose is achieved, the Personal Information will be encrypted and stored in a cloud-based service which may be accessed only by authorised persons. The storage of the Personal Information has the purpose of facilitating internal communication between the Discloser and the Recipient and of allowing internal traceability. For this purpose, the Personal Information will be held for a maximum of 5 years, after this time, without any contact from the Discloser side, the Personal Information will automatically be deleted.

3. The interview may be **recorded** for transcribing purposes only. The audio or video collected shall be encrypted and stored after the transcription is done. The transcription file shall be considered Personal Information and shall be treated accordingly, particularly regarding security of storage.

4. The Recipient shall have the following data protection rights regarding Personal Information: the right of deletion, the right to amend or rectify personal data, the right to object to the use of personal data, the right to restrict processing of data, the right of access, and the right of portability. These rights shall be exercised **in compliance with Regulation 2016/679 (EU)** on the protection of natural persons with regard to the processing of personal data and on the free movement of such data, the General Data Protection Regulation, and in compliance with its articles and recitals. These rights may be exercised upon contact with the Recipient. Survey participants can request the deletion of their data up to the point of manuscript preparation for the publication of the findings, after which de-identified data and information can no longer be deleted. Survey participants can request the deletion of interview recordings, transcripts and personal data held on file at any point in time.

5. The Personal Information will not be published. Unless authorised by the survey participant, no Research Information that could lead to the identification of a specific individual or institution participating in the Survey, will be included in published materials. This means that details that could lead to such identification will be omitted or modified.

6. The Institutional Information may be published in a list of participants in the reporting.

7. The Research Information published shall in no way be linked with the Institutions participating in the survey. Survey results (Research Information) within any subgroup, such as defined by size, technical criteria or geography, will only be published in case there are at least five survey data sets pooled in such subgroup.

8. The Personal, Institutional and Research Information will be provided to the Collaborators in order to achieve the Purpose. The Information shall only be provided to the Collaborators after their signing of a Non-Disclosure Agreement referencing this Statement of Confidentiality.

9. The Discloser of the Information shall be granted access to the Research Information provided by himself with the possibility to revise it within a time period of 4 weeks after the date of the Survey.

10. Nothing in this Statement of Confidentiality will prevent the Recipient from making any disclosure of the Personal, Institutional or Research Information as required by law or by any competent authority.

11. This Statement of Confidentiality is governed by and is to be construed in accordance with Luxembourgish and European law. The Luxembourgish Courts will have non-exclusive jurisdiction to deal with any dispute which has arisen or may arise out of, or in connection with, this Statement of Confidentiality.

Executed by

MEGENO S.A. acting by

Erich Felber, as Managing Director:

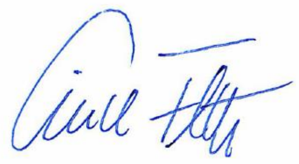

____

Signature of Managing Director
