# Supplementary material for: Genomic Sequencing Capacity, Data Retention, and Personal Access to Raw Data in Europe
Source: Front Genet. 2020 May 6;11:303. doi: 10.3389/fgene.2020.00303 (PMC7218066; doi:10.3389/fgene.2020.00303)
Supplement: Supplementary file 4 [file Data_Sheet_4.PDF]

Supplementary text: Genomic sequencing capacity, data retention and personal access to raw data in Europe

Data privacy, confidentiality and security

Steps taken to ensure the privacy and confidentiality of the institutions and/or respondents were summarised in the confidentiality statement (Supplementary file 4), a copy of which had been provided to all respondents prior to the interviews (section 'Pre-interview communication'). Interviews were scheduled only after a prospective respondent had read and agreed to the conditions described in the confidentiality statement. This information was reiterated at the beginning of the interviews, at which point the respondent was requested, for recording purposes, to provide an explicit verbal consent to participating in the study. Verbal informed consent was solicited in all interviews and was recorded as part of the interview audio/video recordings.

Collaborators and third parties were required to agree in writing to respect confidentiality if they require any data that may reveal the identity of respondents or institutions. When sharing findings of this study publicly, as in the present article, careful steps were taken to anonymise the published data.

Following the completion of data collection, the audio and video recordings were encrypted and archived on an external hard drive, with the encryption keys stored separately. The original audio/video files will be retained for at least five years after publication of the study. Any recording performed without Zoom (section 'Semi structured interviews and recordings'; e.g. phone recording, recording of a personal conversation), was immediately transferred on the Google Suite drive (https://gsuite.google.com/) and deleted from the recording device.

The processed quantitative (section 'Quantitative data analysis and visualisation') and qualitative (section 'Qualitative data analysis') data were masked to avoid potential identification of sequencing institutions and/or respondents. Specifically, the steps to mask quantitative data included i) using independent ID columns in different tables which are ii) independently randomised at least three times, and iii) omission of columns (or data field) that may lead to identification of respondents or institutions. Quotes from qualitative data included in the manuscript are independent of the actual order in which the interviews were carried out or ID columns in the quantitative data (i.e. Supplementary file 2: Tables S1-S6).

Decliners

Five of the 63 contacted institutions declined to undergo an interview for the following reasons:

- Two preferred to answer the interview questions in writing rather than undergoing an oral interview

- One declined because they had no time for an interview
- One declined because they were currently reorganising policies of data storage and access and therefore felt that it was not the best time to contribute to the study
- One did not provide a reason for declining

The rest did not explicitly decline, but either did not respond to our correspondences or failed to schedule an interview.
