# Supplementary material for: Genomic Sequencing Capacity, Data Retention, and Personal Access to Raw Data in Europe
Source: Front Genet. 2020 May 6;11:303. doi: 10.3389/fgene.2020.00303 (PMC7218066; doi:10.3389/fgene.2020.00303)
Supplement: Supplementary file 5 [file Data_Sheet_5.PDF]

2019

SURVEY ON GENOMIC SEQUENCING IN EUROPEAN HEALTHCARE INSTITUTIONS

Full Version

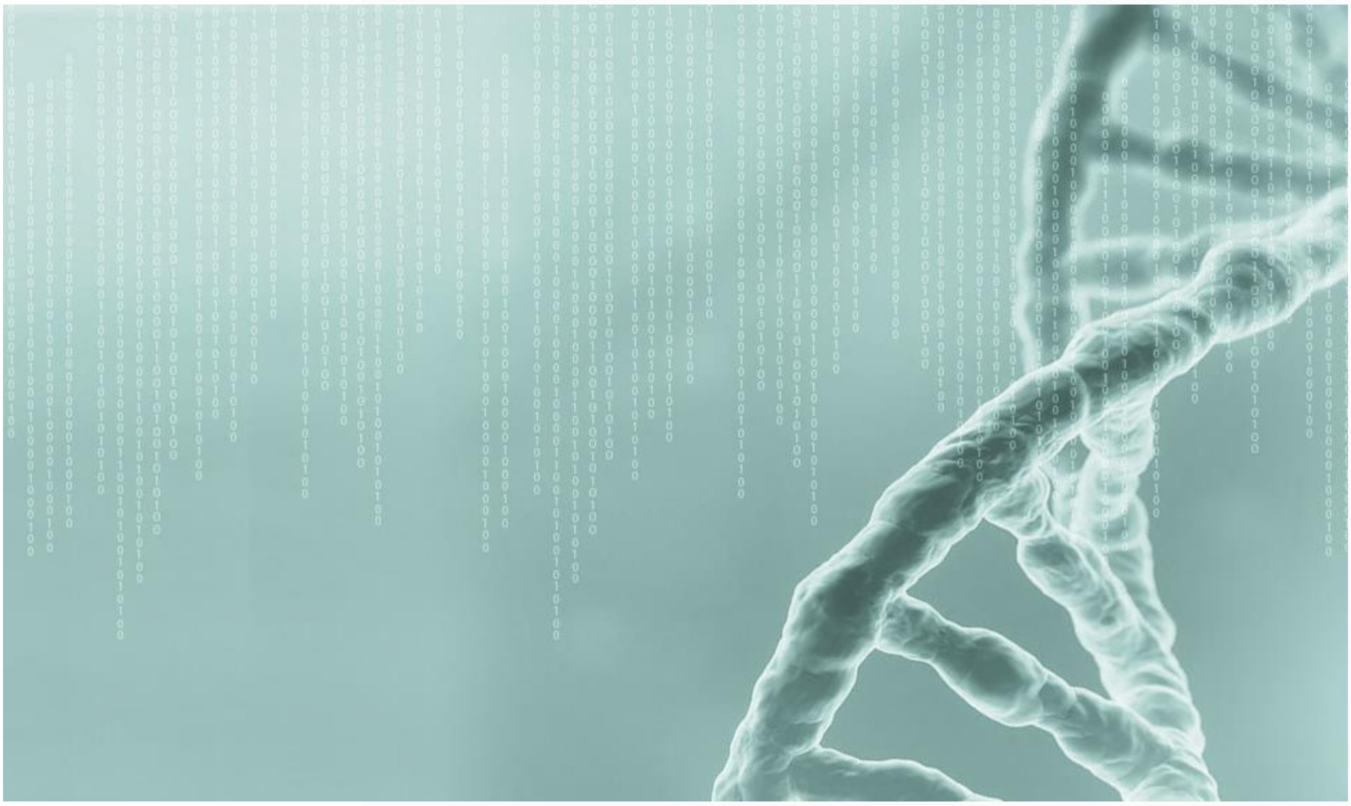

PREPARED BY MEGENO S.A.

www.megeno.com
+352 20 60 63 101
info@megeno.com

6A, AVENUE DES HAUTS-FOURNEAUX
L-4362 ESCH-SUR-ALZETTE
LUXEMBOURG

The Survey

MeGeno is conducting a Europe-wide survey specifically aimed at healthcare and research institutions that perform and interpret human whole genome/exome sequencing.

The Goal

The survey will collect information to assess current policies for whole genome/exome sequence data retention and access. We also cover aspects of organisational structure and technical capabilities. We aim to understand how emerging ethical implications and legal requirements are being implemented by European sequencing and healthcare institutions.

Your Contribution

Your participation and input is very important to us due to the currently limited number of European institutions generating whole genome/exome data. Furthermore, it helps us to understand current practices and unmet needs related to data retention and access policies. You and your institution will remain anonymous in any reporting.

Your Benefits

Your responses will be returned to you for review prior to the analysis and will remain confidential. Subsequently, you will receive summary results of the survey which will provide you with a deep insight into data retention and access policies implemented across Europe.

Module Overview

1. Personal profile
2. Organisational structure
3. Sequencing capacity
4. Data storage capacity
5. Data access policy
6. Data access requests

You may skip questions/modules outside the scope of your activities.

Survey on Genomic Sequencing in European Healthcare Institutions

<u>We would like to kindly remind you that this survey is focused only on whole genome and whole exome sequencing. We are not including gene panels, etc. in the study.</u>

Module 1: Personal Profile

**Please, tell us about your position, functions and a little about your background:**	

Module 2: Organisational Structure

1. Type of institution

Do you have a sequencing facility in your institution?
a. Yes
b. No
c. Other, please specify:

Do you serve clinical purposes?
a. Yes
b. No
c. Other, please specify:

Do you have any ongoing research, which involves human whole genome or whole exome sequencing?
a. Yes
b. No
c. Other, please specify:

Are you part of any consortium?
a. Yes
b. No
c. Other, please specify:

Are you a leader of any consortium?
a. Yes
b. No
c. Other, please specify:

2. Governance

Are you a public or private institution?
a. Public
b. Private
c. Other, please specify:

3. Funding

Your funding is:
a. Public
b. Private
c. Other, please specify:

4. Number of employees

Module 3: Sequencing Capacity

1. When did you start human whole genome/exome sequencing? Please, specify the year for both, WES and WGS.

2. What type of sequencing platform(s) do you operate for whole human genome/exome sequencing? (Fill in the table below)

Platform	Number of units (to date)	Purchase Year	Number of units (future)
Illumina NextSeq 500			
Illumina NextSeq 550/550Dx			
Illumina HiSeq 2000			
Illumina HiSeq 2500			
Illumina HiSeq 3000			
Illumina HiSeq 4000			

Illumina HiSeq X			
Illumina NovaSeq 6000			
Other(s), please specify:			

3. Are you planning on expanding your human whole genome/exome sequencing capacity? (Fill in the table above if applicable)

a. Yes
b. No plans for expansion
c. I do not know

4. Which disease areas do you cover in the context of whole genome/exome sequencing?

Whole genome	Whole exome
a. Cancer<br>b. Rare disease<br>c. Healthy controls (trios, etc.)<br>d. Other(s), please specify:	a. Cancer<br>b. Rare disease<br>c. Healthy controls (trios, etc.)<br>d. Other(s), please specify:

5. How deep do you sequence both, genomes and exomes? Please, indicate the coverage.

6. How many people have you sequenced to date? Additionally, please provide breakdown estimates for the years of 2017, 2018 and 2019.

Data type/Year	Individuals sequenced			
	**to date (total)**	**2017**	**2018**	**2019**
Whole genomes				
Whole exomes				

Module 4: Data Storage Capacity

1. In the whole genome/exome data processing chain (from BCL to VCF/gVCF), **which of the files do you store**?

a. BCL
b. FASTQ
c. BAM
d. CRAM
e. VCF
f. gVCF
g. Other(s), please specify:

2. Do you store these files on-site or externally (e.g. National Computer System, etc.)**?**

3. Is there any specific reason why you store these particular files?

4. Compression applied

Compression/File type	BCL	FASTQ	BAM	Other
gzip/gz				
bzip				
bgzip				
bzip2				
CRAM (for BAM format)				
PetaGene (commercial)				
GeneFormics				
GeneAlice				
Other(s), please specify:				
No compression applied				
I do not know				

5. How many whole genome/exome sequencing data sets do you store/plan to store?

Data type/Year	Number of individuals' data stored			
	**to date (total)**	**2017**	**2018**	**2019**
WGS				
WES				

6. Please, indicate the size of one raw file (FASTQ) in gigabytes.

Module 5: Data Access Policy

1. Does your institution have a data retention policy for whole genome/exome sequencing data?

a. Yes
b. No
c. I do not know

if yes

a. **Is this approach based on a specific law/policy?**

b. **If possible, what is the reason?**

c. **Is it included in your consent?**

- Yes
- No, then **why is it not** included?
- I do not know

if no

a. Why is there no data retention policy?

2. Does your institution have data access policy for whole genome/exome sequencing data specifically geared towards sequenced individuals and/or their physicians?

a. Yes
b. No
c. I do not know

if yes

a. **Is this approach based on a specific law/policy?**

b. **If possible, what is the reason?**

c. **Is it included in your consent?**

- Yes
- No, then why is it not included?
- I do not know

if no

a. **Why is there no data access policy covering the aforementioned parties?**

3. Is your consent form publicly available?

a. Yes
b. No
c. I do not know

4. What changes were implemented by your institution based on GDPR, with regards to whole genome/exome sequencing?

Module 6: Data Access Requests

1. To date, have you/your institution/your collaborators ever been requested access to whole genome/exome data by the sequenced individuals and/or their physicians?

a. Yes
b. No
c. I do not know

IF YES

a. Who requested for the data?

b. How was it communicated? (point of contact, channel)

c. What exactly was asked to be accessed? (Raw data, processed data, interpreted findings)

d. Was it successfully carried out?

Yes	i. Who authorised the access?	No	i. Why were you unable to provide access?
	ii. How was the authorisation processed?		ii. How would you respond to such a request?
	iii. How was the process carried out?		iii. What privacy and/or security measures would you employ when providing this access?
	iv. What privacy and/or security measures do you employ when providing this access?		

if no	**a. Is your organisation prepared/preparing for such potential requests?**			
	Yes	i. How are they prepared/preparing for it?	No	i. How would you or your institution respond to such a request?
		ii. What privacy and/or security measures do you employ when providing this access?		ii. What privacy and/or security measures would you employ when providing this access?
